# Metabolic Mediation of the Association Between Hyperandrogenism and Paratubal Cysts in Polycystic Ovary Syndrome: A Structural Equation Modeling Approach

**DOI:** 10.3390/jcm14155545

**Published:** 2025-08-06

**Authors:** Jin Kyung Baek, Chae Eun Hong, Hee Yon Kim, Bo Hyon Yun

**Affiliations:** 1 Department of Obstetrics and Gynecology, Severance Hospital, Yonsei University College of Medicine, Seoul 03722, Republic of Korea; 2 Department of Medicine, Yonsei University College of Medicine, Seoul 03722, Republic of Korea

**Keywords:** polycystic ovary syndrome, hyperandrogenism, paratubal cysts, insulin resistance, metabolic syndrome

## Abstract

**Objectives**: Paratubal cysts (PTCs) are embryological remnants and are potentially hormonally responsive. Since hyperandrogenism (HA) is representative of polycystic ovary syndrome (PCOS), we examined whether biochemical hyperandrogenism is associated with PTCs in women with PCOS and if body mass index (BMI) and insulin resistance (IR) mediate this association. **Methods**: This retrospective study included 577 women diagnosed with PCOS at a tertiary academic center from 2010 to 2018. Clinical data included age at diagnosis, BMI, and diagnoses of hypertension, non-alcoholic fatty liver disease, and metabolic syndrome. Laboratory measures included total testosterone, sex hormone-binding globulin, anti-Müllerian hormone, luteinizing hormone, fasting glucose, insulin, and triglycerides (TG). Derived indices included a free androgen index (FAI), homeostasis model assessment of insulin resistance (HOMA-IR), and fasting glucose-to-insulin ratio. PTCs were identified through imaging or surgical findings. Structural equation modeling (SEM) assessed direct and indirect relationships between FAI, BMI, HOMA-IR, and PTCs, while adjusting for diagnostic age. **Results**: PTCs were identified in 2.77% of participants. BMI, FAI, TG, and IR indices were significantly higher for women with PTCs than those without PTCs. SEM revealed significant indirect effects of FAI on PTCs via BMI and HOMA-IR. The direct effect was negative, resulting in a non-significant total effect. A sensitivity model using HOMA-IR as the predictor showed a significant direct effect on PTCs without mediation via FAI. **Conclusions**: Biochemical HA may influence PTC development in PCOS through metabolic pathways, establishing the need to consider metabolic context when evaluating adnexal cysts in hyperandrogenic women.

## 1. Introduction

Polycystic ovary syndrome (PCOS) is a heterogeneous endocrine disorder characterized by hyperandrogenism (HA), ovulatory dysfunction, and polycystic ovarian morphology [1,2]. Beyond reproductive implications, PCOS is associated with metabolic disturbances, including obesity, insulin resistance (IR), and increased cardiometabolic risk [3,4,5,6,7,8]. A well-documented pathogenic loop connects IR and HA in which insulin amplifies ovarian androgen production, while HA worsens insulin sensitivity by establishing a vicious cycle that promotes long-term metabolic complications [9,10].

Paratubal cysts (PTCs) are benign adnexal lesions located adjacent to the fallopian tubes and ovaries, which are thought to originate from remnants of the mesonephric or paramesonephric ducts [11,12,13]. While typically asymptomatic and incidentally discovered, these cysts do not regress on their own and may enlarge over time and/or pose a risk for adnexal torsion [14]. Also, the pathogenesis of PTC remains largely unclear [11,15]. Given their embryological origin, PTCs are suspected to exhibit androgen sensitivity [11,15]. Importantly, biochemical HA in PCOS rarely occurs in isolation; it is tightly intertwined with metabolic dysfunction, including IR and obesity [9,10]. Therefore, we hypothesized that metabolic parameters may serve as downstream mediators of PTC development in hyperandrogenic populations, guiding our decision to focus on these variables in the current study. However, no studies have systematically examined hormonal or metabolic correlates of PTCs, particularly in the context of PCOS.

Considering that PTCs are androgen-sensitive remnants whose size or number may be related to androgen excess, we explored whether phenotypic and endocrine-metabolic disturbances characteristic of PCOS—such as HA, IR, and metabolic syndrome (MetS)—are associated with PTCs presence. Preliminary comparisons between women with and without PTCs revealed significant differences in body mass index (BMI) and IR, suggesting a potential mechanistic link and guiding subsequent hypothesis generation.

To further explore this relationship, we employed structural equation modeling (SEM), which allowed simultaneous estimation of direct and indirect effects between the free androgen index (FAI), BMI, or homeostasis model assessment of insulin resistance (HOMA-IR), and PTCs presence.

## 2. Methods

### 2.1. Study Population

**Figure 1:**
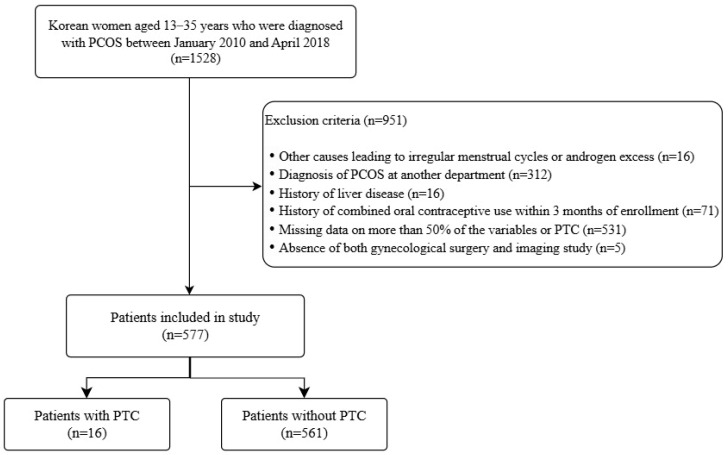
Flow diagram of patient inclusion and exclusion criteria. PCOS, polycystic ovary syndrome; PTC, paratubal cyst.

This retrospective study included Korean women diagnosed with PCOS between January 2010 and April 2018 at Severance Hospital, Seoul, Korea. Adolescents and adults were defined as those aged 10–19 [16] and 20 or older, respectively. PCOS was diagnosed using the 2009 AE-PCOS criteria for adolescents [17] and 2003 Rotterdam criteria for adults [18]. Among 1528 PCOS patients, exclusions were made for alternative causes of HA or oligomenorrhea (e.g., thyroid dysfunction, Congenital adrenal hyperplasia, Cushing’s syndrome), prior hormone or metformin use, insufficient imaging or clinical data, and diagnoses made outside the gynecology department, as detailed in Figure 1. The final sample included 577 patients (Figure 1).

### 2.2. PCOS Diagnosis Criteria

In adults, PCOS diagnosis required two of the following: oligo-anovulation, clinical or biochemical HA, and polycystic ovaries on ultrasound (≥12 follicles or ovarian volume > 10 mL) [18,19]. In adolescents, diagnosis required evidence of HA and persistent oligomenorrhea, excluding other etiologies [17,19].

### 2.3. Clinical and Laboratory Measures

Data collected included age at diagnosis, BMI, and diagnoses of hypertension, non-alcoholic fatty liver disease (NAFLD), and MetS. Hormonal and metabolic profiles were assessed using standard assays for total cholesterol, triglycerides (TG), high-density lipoprotein (HDL), low-density lipoprotein, sex hormone binding globulin (SHBG), anti-Müllerian hormone (AMH), total testosterone (T), dehydroepiandrosterone sulfate (DHEAS), luteinizing hormone, follicle-stimulating hormone, estradiol, fasting glucose to insulin ratio (FGIR), and post-load glucose/insulin (75 g OGTT).

Using electrochemiluminescence (ECLIA; Cobas), serum levels of total T, SHBG, and AMH were measured at the laboratory of our institution. The total T values were converted from ng/mL to nmol/L using a conversion factor of 1 ng/mL = 3.47 nmol/L, as recommended by the manufacturer. FAI was calculated as (total T [nmol/L] × 100)/SHBG [nmol/L]. SHBG < 35 nmol/L and FAI > 4.5% were considered abnormal. HOMA-IR was calculated as [fasting glucose × insulin]/405, with IR defined as HOMA-IR > 3.16 in adolescents and >2.5 in adults [20]. Additionally, FGIR < 7 (adolescents) and <4.5 (adults) were used to define IR [21,22]. Hormonal values were measured during the early follicular phase (day three of the cycle) [23].

In a prospective assessment of the modified Ferriman-Gallwey (mF-G) score conducted by a single investigator on 1010 Korean women, it was determined that a score of six or higher indicated hirsutism within this population. Consequently, in this study, clinical HA was defined as an mF-G score ≥ 6 [24]. NAFLD was diagnosed based on imaging or abnormal liver enzymes reviewed by a hepatologist [25]. MetS was defined per NCEP-ATP III criteria as follows [26]: (1) abdominal obesity, defined as a waist circumference (WC) exceeding 88 cm; (2) elevated TG levels of 150 mg/dL or higher; (3) low HDL cholesterol levels below 50 mg/dL; (4) elevated blood pressure, with readings of 130/85 mmHg or higher; and (5) impaired fasting glucose levels between 110 and 126 mg/dL and/or 2-h postprandial glucose levels ranging from 140 to 199 mg/dL. As WC measurements were not routinely conducted in our study, we used BMI as an alternative measure, using BMI ≥ 25 kg/m^2^ as a surrogate for abdominal obesity in accordance with Asian-specific guidelines [27].

### 2.4. Assessment of PTCs

PTC presence was identified using gynecologic ultrasound, computed tomography, or magnetic resonance imaging and confirmed via imaging or surgical pathology where available [12]. Gynecologic US was performed to confirm symptoms like abnormal uterine bleeding or amenorrhea, while CT and MRI were used for diagnostic purposes in other departments. For MRI and CT images, final identification of PTCs was based on radiologic interpretations from board-certified radiologists. PTCs were identified via transvaginal sonography as well-defined, unilocular cystic structures located adjacent to but separate from the ovary, with a thin outer wall (3 mm), anechoic internal content, and absence of surrounding follicular structures. In most cases, the “split sign” was observed, indicating independent mobility of the cyst and ovary upon probe pressure [28]. These criteria were based on previously established sonographic-pathologic correlations [29]. Cyst size was defined as (width + length)/2. Imaging was performed to evaluate symptoms such as amenorrhea or abnormal bleeding.

### 2.5. Statistical Analysis

Normality of continuous variables was tested using the Shapiro–Wilk test. As no variables were normally distributed, Mann–Whitney U tests were used for group comparisons. Categorical variables were analyzed using the chi-squared test (if all expected cell counts were ≥5); otherwise, Fisher’s exact tests were applied. Among the variables analyzed, only obesity and clinical HA met chi-squared assumptions.

To assess the direct and indirect effects of HA on PTC risk, two SEMs were constructed using the lavaan package (version 0.6-19) in R (version 4.4.2; R Foundation for Statistical Computing, Vienna, Austria) [30]. Model 1 evaluated the effect of FAI on PTC mediated through BMI (FAI → BMI → PTC), and Model 2 evaluated the effect of FAI mediated through insulin resistance (FAI → HOMA-IR → PTC). In both models, diagnostic age was included as a covariate. PTC was coded as binary outcome variable and treated as continuous for SEM approximation, given the small number of cases. Model estimation was conducted using maximum likelihood estimation with robust standard errors. Standardized regression coefficients (β) and *p*-values were used for interpretation.

As the model was saturated due to the small sample size, global model fit statistics were not applicable; thus, interpretation relied on the significance and strength of standardized paths. To enhance the robustness of the mediation analysis, we additionally conducted a non-parametric bootstrapping with 1000 resamples. Confidence intervals for direct and indirect effects were estimated using the bias-corrected and accelerated (BCa) method, which is recommended in small-sample or non-normal data contexts to reduce bias and improve coverage accuracy [31,32].

As a sensitivity analysis, a third model specifying HOMA-IR as the independent variable and FAI as a mediator was additionally tested in order to examine the possibility of reverse causality (see Supplementary Table S1). All analyses were conducted in R version 4.4.2 (R Foundation for Statistical Computing, Vienna, Austria).

## 3. Results

### 3.1. Comparison of Baseline Characteristics with and Without PTCs in Women with PCOS

Among the 577 PCOS patients who met the inclusion criteria, PTC incidence was 2.77% (*n* = 16), comprising five adolescents (4.9%) and eleven adults (2.32%)

Baseline characteristics stratified by PTC presence are presented in Table 1. Women with PTCs were significantly younger than women without PTCs at the time of PCOS diagnosis (*p* = 0.004). BMI (*p* = 0.003), obesity prevalence (*p* = 0.001), and TG levels (*p* = 0.002) were significantly higher in women with PTCs than those of women without PTCs. Moreover, HDL levels were significantly lower in women with PTCs than those in women without (*p* = 0.031). The prevalence of NAFLD (*p* = 0.042) was significantly higher in the PTC group than that in the non-PTC group. Among PCOS women with PTCs, 43.8% met the diagnostic criteria for Mets (≥3 components), compared to 13.7% in group without PTCs. (*p* = 0.004). The significant difference was also found in the distribution of the number of MetS components (*p* = 0.002). Markers of IR, including fasting insulin (*p* = 0.002), FGIR (*p* = 0.005), and HOMA-IR (*p* = 0.002) were significantly higher in women with PTCs than those in women without PTCs. Additionally, hormonal parameters such as SHBG (*p* = 0.014) and FAI (*p* = 0.038) showed statistical differences between groups, whereas the proportion of patients with clinical HA did not (*p* = 0.595).

### 3.2. Characteristics of Women Diagnosed with PTCs

Table 2 summarizes the clinical and diagnostic characteristics of the 16 women diagnosed with PTCs. Among them, 75% (*n* = 12) were classified as obese, 25% (*n* = 4) had NAFLD, and 43.8% (*n* = 7) met the criteria for MetS. Regarding HA, 63% (*n* = 10) of the women had elevated FAI (≥4.5), and 75% (*n* = 12) exhibited clinical signs of HA. Moreover, IR was common: 43.8% (*n* = 7) had low FGIR, and 68.8% (*n* = 11) had high HOMA-IR based on age-specific thresholds. Ten patients (62.5%) were diagnosed through surgical exploration, while the remaining six were diagnosed via ultrasonography. Mean PTC size was 6.47 cm (range, 2.0–12.5 cm) and 2.67 cm (range, 1.65–3.7 cm) in surgically-confirmed and ultrasound-only cases, respectively. The overall average size was 5.04 cm (range, 1.65–12.5 cm).

### 3.3. Direct and Indirect Relationship Between Androgen Levels and PTC Occurrence

To evaluate both direct and indirect pathways through which androgen excess may influence the PTC presence, an SEM approach was employed. In model 1, FAI was specified as the independent variable, BMI as a mediator, and PTC as the outcome variable. Diagnostic age (DxAge) was included as a covariate. SEM results (Table 3) indicated that FAI had a significant positive effect on BMI (standardized β = 0.470, *p* < 0.001), and BMI was positively associated with PTC occurrence (standardized β = 0.174, *p* = 0.006). FAI showed a significant negative direct association with PTCs (standardized β = −0.092, *p* = 0.015). Although the indirect effect of FAI on PTCs via BMI was statistically significant (standardized β = 0.082, *p* = 0.010), the total effect was not (standardized β = −0.010, *p* = 0.705). Robustness was further evaluated using non-parametric bootstrapping with 1000 resamples and bias-corrected and accelerated confidence intervals. The indirect effect of FAI on PTC via BMI remained statistically significant (unstandardized β = 0.003, 95% BCa CI [0.001, 0.005], *p* = 0.013; standardized β = 0.081), as did the direct effect (β = −0.003, 95% CI [−0.006, −0.001], *p* = 0.019; standardized β = −0.092). The total effect was non-significant (β = −0.000, *p* = 0.708). These findings support the stability of the mediation pathway.

Given the established pathophysiological link between IR and HA in women with PCOS, we conducted an SEM analysis to assess whether FAI affects the risk of PTCs directly or indirectly through HOMA-IR (Table 4, model 2). DxAge was included as a covariate. The model showed that FAI had a significant positive effect on HOMA-IR (standardized β = 0.378, *p* < 0.001), and HOMA-IR had a significant direct effect on PTC occurrence (β = 0.156, *p* = 0.042). The direct effect of FAI on PTCs did not reach statistical significance (β = −0.069, *p* = 0.098), while the indirect effect of FAI on PTCs via HOMA-IR was statistically significant (β = 0.059, *p* = 0.049). The total effect of FAI on PTCs remained non-significant (β = −0.010, *p* = 0.723).

## 4. Discussion

This study is based on the clinical observation that HA is a common endocrine feature in women with PCOS, while building upon our hypothesis that elevated androgen levels might be associated with the PTC presence. To date, this association has not been systematically investigated. Thus, we examined whether biochemical HA was directly or indirectly related to PTC occurrence in women with PCOS. Through SEM, we found that FAI was positively associated with both BMI and IR, which in turn were positively associated with PTCs. However, the direct association between FAI and PTCs was negative, resulting in a non-significant total effect. These findings suggest that metabolic factors such as BMI and IR may mediate the relationship between HA and PTCs, with potentially opposing effects from direct and indirect pathways contributing to the overall complexity of this association.

Previous studies have proposed that PTCs are androgen-sensitive remnants, based on their embryological origin from mesonephric or paramesonephric structures [13,15]. A recent study investigating the association between PTC formation and PCOS reported that adolescent girls with a PTC were more likely to be overweight and to have or develop PCOS compared to age-matched controls [28]. These findings are consistent with our results and further support the notion that PTCs may be associated with underlying metabolic and hormonal dysregulation. Our findings suggest that systemic androgen excess in PCOS may not directly cause PTC formation but may do so indirectly by promoting adiposity and IR. This aligns with the known pathogenic cycle in PCOS, where HA and IR reinforce one another and contribute to a metabolic environment conducive to structural changes in reproductive tissues [6,7,9,10,27,33].

The presence of opposing effects—negative direct and positive indirect associations—raises the possibility of threshold-dependent or context-specific effects of androgen excess. Additionally, compensatory or protective mechanisms may modulate the influence of FAI on PTC formation. These findings highlight the importance of considering both hormonal and metabolic factors when evaluating gynecologic structural changes in PCOS. To date, there are no established biological mechanisms or empirical studies that explain a negative direct association between FAI and PTCs. Therefore, this result should be considered exploratory and interpreted with caution until further mechanistic studies can validate or refute this finding

To our knowledge, our study is the first to investigate the association between HA and PTCs using a path analysis framework that utilizes SEM models to evaluate both FAI- and HOMA-IR-based pathways. Additionally, we tested a reverse pathway model as a sensitivity analysis in which HOMA-IR was treated as the independent variable and FAI as the mediator (Model 3; Supplementary Table S1). In Model 3 (HOMA-IR → FAI → PTC), IR demonstrated a significant direct effect on PTC occurrence, while the indirect effect via FAI was not statistically significant. These findings suggest that while HOMA-IR may influence androgen levels, its effect on PTC development is likely exerted directly rather than through elevated androgens. The robustness of the direct effect across different model structures supports the potential mechanistic role of IR in PTC pathogenesis.

While previous studies have suggested that PTCs may correlate with obesity or hormonal changes in adolescents [28,34,35], none have evaluated causal pathways in a PCOS-specific population. Our findings offer novel insights into how endocrine-metabolic disruptions may facilitate the pathogenesis of PTCs.

However, our study has notable limitations. First, the retrospective and cross-sectional design of our study precludes any causal inferences between biochemical HA and PTCs. Although SEM allows for the estimation of hypothesized pathways, it does not establish temporal or causal relationships. Therefore, all associations observed in this study should be interpreted as correlational. Second, PTCs identification was based on imaging or operative findings, which could lead to low sensitivity, particularly in the case of small or asymptomatic cysts [36,37,38,39]. Cysts smaller than 10–12 mm in diameter could have been underdetected due to the resolution limits of ultrasound, especially in the absence of MRI or surgical confirmation. Third, the limited number of PTCs cases might have reduced the statistical power of some analyses. Since the SEM were saturated (zero degrees of freedom), global model fit (e.g., χ^2^, Root Mean Square Error of Approximation, Comparative Fit Index) could not be computed. As a result, model evaluation relied solely on the significance and direction of individual path coefficients, rather than overall fit. To mitigate this limitation, we have supported BCa confidence intervals using bootstrap-based estimation of indirect effects, but we acknowledge that these approaches do not fully compensate for the low statistical power inherent in rare-event analysis [31,32]. As such, the current results should be interpreted with caution and should be considered exploratory. Last, selection bias cannot be excluded because this cohort was drawn from a single tertiary hospital.

Future studies should incorporate prospective imaging protocols with higher diagnostic accuracy to better capture the prevalence and natural history of PTCs. Additionally, comparisons with age- and BMI-matched women without PCOS can help clarify whether PTCs are a distinctive structural manifestation of the PCOS metabolic milieu. Furthermore, it would be worthwhile to examine whether cyst dimensions show stronger correlation with androgen level, and to explore the potential mechanistic link between metabolic dysfunction and HA in the pathogenesis of PTCs.

## 5. Conclusions

In women with PCOS, biochemical HA may contribute to PTCs development, potentially through metabolic intermediaries such as obesity and IR. These findings underscore the importance of considering metabolic context when evaluating structural gynecologic abnormalities in hyperandrogenic populations. However, as this study lacks a control group, the observed associations are limited to PCOS patients. It remains unclear whether the prevalence of PTCs is lower in women without PCOS. Given the high prevalence of PCOS and its associated metabolic complications, clinicians should be aware of potential adnexal structural changes that may arise indirectly from hormonal disturbances. Future prospective studies including non-PCOS controls and larger multicenter cohorts are essential to validate these associations and clarify the pathophysiologic mechanisms underlying PTCs formation.

## Figures and Tables

**Table 1:** Baseline characteristics of women with PCOS stratified by paratubal cyst presence.

Variables	With PTCs (*n* = 16)	Without PTCs (*n* = 561)	*p*-Value
Age at PCOS Dx (year)	20.5 [17.0;23.5]	24.0 [21.0;29.0]	**0.004**
BMI (kg/m^2^)	28.0 [24.9;31.3]	22.4 [19.9;26.4]	**0.003**
Obesity (*n*)	12 (75%)	183 (32.6%)	**0.001**
Total cholesterol (mg/dL)	195.0 [165.0;225.5]	180.0 [161.0;204.0]	0.191
Triglycerides (mg/dL)	146.5 [87.5;207.0]	81.5 [58.0;121.0]	**0.002**
HDL (mg/dL)	46.0 [37.0;61.0]	57.0 [47.0;67.5]	**0.031**
LDL (mg/dL)	115.2 [95.5;149.7]	101.0 [85.0;121.8]	0.127
Fasting glucose (mg/dL)	91.0 [87.0;103.0]	91.0 [86.0;96.0]	0.443
2-h 75 g GTT glucose (mg/dL)	110.0 [97.5;140.0]	104.0 [89.0;124.0]	0.149
Fasting insulin (µU/mL)	15.9 [11.4;24.1]	8.9 [5.4;14.3]	**0.002**
2-h 75 g GTT insulin (µU/mL)	77.6 [45.4;150.6]	51.1 [30.5;94.0]	0.081
FGIR	6.7 [3.7;9.0]	10.1 [6.5;16.8]	**0.005**
HOMA-IR	3.6 [2.5;8.3]	2.0 [1.2;3.3]	**0.002**
Total testosterone (ng/dL)	34.0 [25.8;59.4]	41.5 [30.7;54.4]	0.636
SHBG (nmol/L)	22.9 [18.9;39.4]	44.9 [26.7;70.4]	**0.014**
FAI (%)	5.0 [4.2;6.8]	3.2 [1.7;6.2]	**0.038**
DHEAS (µU/dL)	205.0 [153.0;326.0]	219.5 [164.0;289.0]	0.728
LH (mIU/mL)	10.2 [7.7;13.0]	8.8 [5.5;13.9]	0.599
FSH (mIU/mL)	5.8 [5.0;6.6]	6.2 [5.0;7.3]	0.375
Estradiol (pg/mL)	39.5 [28.0;73.0]	45.0 [29.0;62.0]	0.808
AMH (ng/mL)	8.6 [5.8;11.8]	11.0 [7.0;16.8]	0.088
Clinical HA (*n*)	12 (75%)	370 (66.0%)	0.627
NAFLD (*n*) ^†^	4 (25%)	46 (8.2%)	**0.042**
HTN (*n*) ^†^	1 (6.2%)	37 (6.6%)	1.000
Number of MetS components (*n*) ^†^			
0	2 (12.5%)	269 (48%)	
1	4 (25%)	138 (24.6%)	
2	3 (18.8%)	77 (13.7%)	
≥3	7 (43.8%)	77 (13.7%)	**0.00** **4**
Overall			**0.002**

Values are presented as the median [Q1;Q3] or numbers (%). Bolded *p*-values indicate statistical significance at *p* < 0.05. ^†^ Fisher’s exact tests were used when the expected frequency in any cell was <5; otherwise, chi-squared tests were applied. PCOS, polycystic ovary syndrome; BMI, body mass index; HDL, high-density lipoprotein; LDL, low-density lipoprotein; GTT, glucose tolerance test; FGIR, fasting glucose insulin ratio; HOMA-IR, homeostasis model assessment of insulin resistance; SHBG, sex hormone-binding globulin; FAI, free androgen index; DHEAS, dehydroepiandrosterone sulfate; LH, luteinizing hormone; FSH, follicle-stimulating hormone; AMH, anti-Müllerian hormone; HA, hyperandrogenism; NAFLD, non-alcoholic fatty liver disease; HTN, hypertension; MetS, metabolic syndrome.

**Table 2:** Clinical and metabolic characteristics of women with PCOS diagnosed with PTCs (*n* = 16).

	PTC Cases (%)
Age at PCOS diagnosis	
Adolescent	5 (31)
Adult (≥20 years old)	11 (59)
Method of diagnosis	
Surgery	10 (63)
ultrasonography	6 (38)
Obesity	12 (75)
FAI > 4.5	10 (63)
Clinical hyperandrogenism	12 (75)
Metabolic syndrome	7 (44)
Insulin resistance	
FGIR < 7 for adolescents, <4.5 for adults	7 (44)
HOMA-IR > 3.16 for adolescents, >2.5 for adults	11 (59)

Values are presented as numbers (percentages of a total of 16). PCOS, polycystic ovary syndrome; PTC, paratubal cyst; FAI, free androgen index; FGIR, fasting glucose to insulin ratio; HOMA-IR, homeostasis model assessment of insulin resistance.

**Table 3:** Structural equation model assessing the effect of FAI on paratubal cysts mediated by BMI in women with PCOS (Model 1: FAI → BMI → PTC).

Pathway	Effect Type	Standardized Coefficient	*p*-Value
FAI → BMI	Direct	0.47	**<0.001**
BMI → PTC	Direct	0.174	**0.006**
FAI → PTC	Direct	−0.092	**0.015**
FAI → BMI → PTC	Indirect	0.082	**0.010**
FAI → PTC (Total effect)	Total	−0.01	0.705

Bolded *p*-values indicate statistical significance at *p* < 0.05. PCOS, polycystic ovary syndrome; BMI, body mass index; PTC, paratubal cyst; FAI, free androgen index.

**Table 4:** Structural equation model assessing the effect of FAI on paratubal cysts mediated by HOMA-IR in women with PCOS (Model 2: FAI → HOMA-IR → PTC).

Pathway	Effect Type	Standardized Coefficient	*p*-Value
FAI → HOMA-IR	Direct	0.378	**<0.001**
HOMA-IR → PTC	Direct	0.156	**0.042**
FAI → PTC	Direct	−0.069	0.098
FAI → HOMA-IR → PTC	Indirect	0.059	**0.049**
FAI → PTC (Total effect)	Total	−0.01	0.723

Bolded *p*-values indicate statistical significance at *p* < 0.05. PCOS, polycystic ovary syndrome; PTC, paratubal cyst; FAI, free androgen index; HOMA-IR, homeostasis model assessment of insulin resistance.

## Data Availability

Original data generated and analyzed during this study are included in this published article or in the data repositories listed in the references.

## Supplementary Materials

The following supporting information can be downloaded at: https://www.mdpi.com/article/10.3390/jcm14155545/s1, Table S1: Structural equation model assessing the effect of HOMA-IR on paratubal cysts mediated by FAI in women with PCOS (Model 3: HOMA-IR → FAI → PTC).
