# Supplementary material for: Metabolic Mediation of the Association Between Hyperandrogenism and Paratubal Cysts in Polycystic Ovary Syndrome: A Structural Equation Modeling Approach
Source: J Clin Med. 2025 Aug 6;14(15):5545. doi: 10.3390/jcm14155545 (PMC12347165; doi:10.3390/jcm14155545)
Supplement: Supplementary file 1 [file jcm-14-05545-s001.zip › jcm-3736152-supplementary.pdf]

Supplementary Table S1. Structural equation model assessing the effect of HOMA-IR on paratubal cysts mediated by FAI in women with PCOS (Model 3: HOMA-IR → FAI → PTC)

Pathway	Effect Type	Standardized Coefficient	P-value
HOMA-IR → FAI	Direct	0.351	**< 0.001**
FAI → PTC	Direct	-0.069	0.098
HOMA-IR → PTC	Direct	0.156	**0.042**
HOMA-IR → FAI → PTC	Indirect	-0.024	0.124
HOMA-IR → PTC (Total effect)	Total	0.132	**0.046**

PCOS, polycystic ovary syndrome; PTC, paratubal cyst; FAI, free androgen index; HOMA-IR, homeostasis model assessment of insulin resistance. Bolded *p*-values indicate statistical significance at $p < 0.05$.
